# Ultrasensitive Linear Capacitive Pressure Sensor with Wrinkled Microstructures for Tactile Perception

**DOI:** 10.1002/advs.202206807

**Published:** 2023-03-15

**Authors:** Chunyu Lv, Chengcheng Tian, Jiashun Jiang, Yu Dang, Yang Liu, Xuexin Duan, Quanning Li, Xuejiao Chen, Mengying Xie

**Affiliations:** ^1^ State Key Laboratory of Precision Measuring Technology and Instrument School of Precision Instrument and Opto‐electronics Engineering Tianjin 300072 P. R. China; ^2^ College of Artificial Intelligence Nankai University Tianjin 300350 P. R. China

**Keywords:** hardness discrimination, physiological signal monitoring, pressure sensors, soft pneumatic finger, spontaneous microstructures, tactile perception

## Abstract

Ultrasensitive flexible pressure sensors with excellent linearity are essential for achieving tactile perception. Although microstructured dielectrics have endowed capacitive sensors with ultrahigh sensitivity, the compromise of sensitivity with increasing pressure is an issue yet to be resolved. Herein, a spontaneously wrinkled MWCNT/PDMS dielectric layer is proposed to realize the excellent sensitivity and linearity of capacitive sensors for tactile perception. The synergistic effect of a high dielectric constant and wrinkled microstructures enables the sensor to exhibit linearity up to 21 kPa with a sensitivity of 1.448 kPa^−1^ and a detection limit of 0.2 Pa. Owing to these merits, the sensor monitors subtle physiological signals such as various arterial pulses and respiration. This sensor is further integrated into a fully multimaterial 3D‐printed soft pneumatic finger to realize material hardness perception. Eight materials with different hardness values are successfully discriminated, and the capacitance of the sensor varies linearly (*R* ^2^ > 0.975) with increasing hardness. Moreover, the sensitivity to the material hardness can be tuned by controlling the inflation pressure of the soft finger. As a proof of concept, the finger is used to discriminate pork fats with different hardness, paving the way for hardness discrimination in clinical palpation.

## Graphical abstract

A capacitive tactile sensor based on a spontaneously wrinkled dielectric layer realizes ultrahigh sensitivity and linearity over a broad sensing range for physiological health monitoring. Furthermore, integrated with a multimaterial 3D‐printed soft pneumatic finger, the sensor achieves linear material hardness perception, and a tunable sensitivity to hardness is also obtained by controlling the inflation pressure of the finger.

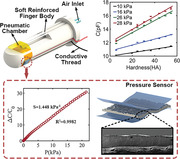

## 1 Introduction

Tactile perception is important in detecting physical interactions, and has shown great promise in wearable electronics, intelligent robotics and prosthetics.^[^ ^1^, ^2^, ^3^ ^]^ Ultrasensitive flexible capacitive pressure sensors are essential components of tactile sensing. Enormous effort has been devoted to improving the performance of pressure sensors.^[^ ^1^ ^]^ Nevertheless, most reported sensors suffer from nonlinear sensitivity with increasing pressure, which leads to compromised resolution and redundant post‐data processing and conversion.^[^ ^4^ ^]^ It is highly desirable to develop a pressure sensor with both high sensitivity and excellent linearity over a broad pressure range for practical applications.

Ultrahigh sensitivity and linearity, two of the most critical prerequisites for accurate tactile sensing, are still outstanding challenges for pressure sensors. Currently, embedding microstructured dielectric materials with a low Young's modulus to obtain high compressibility has proven to be one of the most effective methods for improving sensitivity.^[^ ^5^, ^6^, ^7^, ^8^ ^]^ These microstructures are often engineered using complex and expensive technologies such as photolithography and etching. Additionally, some studies have demonstrated that an increase in the dielectric constant can effectively improve the sensitivity of the sensors.^[^ ^9^, ^10^ ^]^ These dielectrics with high permittivity are commonly fabricated by mixing high‐dielectric‐constant particles or conductive fillers into the polymer matrix.^[^ ^11^, ^12^ ^]^ However, the above two methods play a role mainly in a narrow low‐pressure range. When the pressure increases, the sensitivity drops dramatically, which leads to poor linearity and limits the practical applications. Therefore, recent studies have focused not only on ultrahigh sensitivity, but also on improving the linearity of pressure sensors. Zhou et al. studied the structural design and proposed a dielectric layer with hierarchical microdomes to achieve a stepwise contact between the dielectric layer and electrode, which led to outstanding linearity (*R* ^2^ = 0.99) and a sensitivity of 0.065 kPa^−1^ in an ultra‐broad pressure range.^[^ ^13^ ^]^ In addition, a method to improve linearity by tuning the filler contour and concentration has also been demonstrated. For example, when common spherical nickel particles were replaced by spiky nickel particles as filler materials, the hybrid composite exhibited extraordinary linearity (*R* ^2^ = 0.999) and the pressure range was up to 1.7 MPa.^[^ ^14^ ^]^ However, the sensitivity was only 0.0046 kPa^−1^. Therefore, although the above approaches enable sensors with either ultrahigh sensitivity or excellent linearity, simultaneously realizing high sensitivity and linearity for pressure sensors remains a great challenge.

Ultrasensitive and linear pressure sensors have great potential for tactile perception such as health monitoring and hardness discrimination. Pressure sensors capable of reliable real‐time monitoring of arterial pulses^[^ ^15^ ^]^ have attracted attention because of their importance in cardiovascular disease diagnosis and noninvasive usage. To monitor such subtle physiological signals, sufficiently high sensitivity and resolution are prerequisites for the sensors. Additionally, the tactile feedback of contact pressure and the information of the object materials^[^ ^16^ ^]^ have been promoting safe and dexterous manipulations. Hardness is a material mechanics‐related physical property, and its significance in guaranteeing the safety of objects has been demonstrated, especially in agricultural picking and minimally invasive surgery.^[^ ^17^, ^18^ ^]^ In addition, embedding the tactile sensors capable of hardness discrimination into a silicon fingertip can further improve the safety of manipulations.^[^ ^19^ ^]^ Moreover, soft pneumatic robots play an important role in decreasing the damage of substances from traditional rigid grippers, thus improving the security of manipulations.^[^ ^20^ ^]^ Therefore, inspired by novel soft robotics, combining pressure sensors with a soft pneumatic chamber to realize hardness tactile perception is a practical and promising method.

In this work, we present an ultrasensitive flexible capacitive pressure sensor with a broad linearity range based on a spontaneously wrinkled multiwall carbon nanotube/polydimethylsiloxane (MWCNT/PDMS) composite dielectric layer. Compared with complex fabrication technologies, the dielectric composite film was fabricated by a simple spin‐coating process and wrinkled microstructures were formed owing to the aggregation of MWCNT. The combination of these wrinkled microstructures and percolating MWCNT fillers contributes to ultrahigh sensitivity and linearity. This was achieved by tuning the MWCNT concentration. The proposed sensor with 2.6 wt% MWCNTs can achieve a high sensitivity of 1.448 kPa^−1^ and excellent linearity (*R* ^2^ = 0.9982) in the pressure range of 0.005–21 kPa. The sensor also showed a fast response time, good durability, and could even detect pressures as low as 0.2 Pa. Furthermore, the concept of pressure sensors has numerous merits. The feasibility of tactile perception applications in a different real‐life context is demonstrated, especially for continuous health monitoring by recording various artery pulsations and respiration. In addition, a flexible pressure sensor was attached to a 3D printed pneumatic soft finger to discriminate eight materials with different hardness values. The results show a linear relationship between the capacitance change and material hardness (covering the range of 5.5 HA to 54.5 HA), and the sensitivity to hardness can be enhanced by increasing the inflation pressure. Moreover, we successfully discriminated between the hardness of soft fat and stiffened fats of pork, which paves the way for application in clinical palpation.

## 2 Results and Discussion

### 2.1 Sensor Fabrication and Characterization

**Figure 1:**
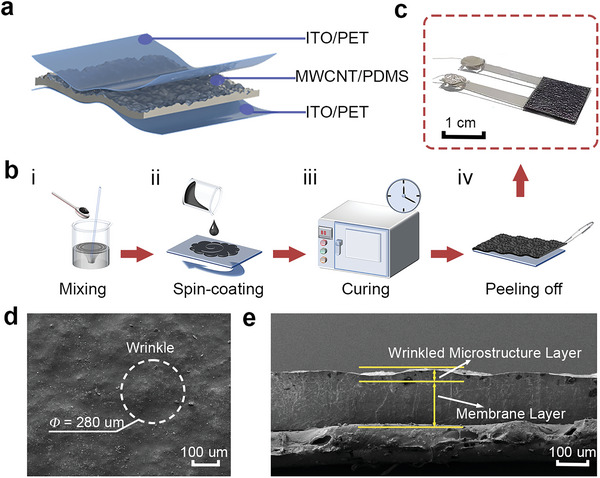
Proposed capacitive sensor. a) Schematic of the capacitive sensor and photograph of the sensor. b) Schematic illustration of the fabrication process. i) Mixing the MWCNT into PDMS matrix, ii) spin‐coating the MWCNT/PDMS mixture, iii) curing the MWCNT/PDMS membrane in an oven, and iv) releasing the membrane from the substrate. c) Photograph of our capacitive sensor. d) SEM photograph of the surface of the MWCNT/PDMS dielectric layer. e) Cross‐section SEM image of the dielectric layer.

**Figure** **1**a shows a schematic of the flexible pressure sensor, where the MWCNT/PDMS dielectric layer was sandwiched between two indium tin oxide/polyethylene terephthalate (ITO/PET) flexible electrodes. The key component of this sensor is a microstructured dielectric layer, which is fabricated by simply spin‐coating a mixture of MWCNT and PDMS. The fabrication process of the flexible pressure sensor is shown in Figure 1b. First, the MWCNT powder was uniformly dispersed into a hexane solution via a 20 min ultrasonic treatment. The PDMS precursor was then poured and magnetically stirred to ensure a homogeneous distribution. The as‐prepared mixture was spin‐coated onto a clean glass substrate at 250 rpm for 20 s and then placed in a vacuum desiccator to remove residual hexane. Subsequently, the MWCNT/PDMS composite film was cured at 90 °C for 1 h. Finally, the prepared MWCNT/PDMS dielectric layer was cut into 1 × 1 cm^2^ pieces and sandwiched between two identical ITO/PET electrodes to form a capacitive sensor, as shown in Figure 1c. In this work, to systematically investigate the impact of MWCNT fillers on the dielectric constant and the sensor performance, four different MWCNT weight ratios, that is, 1 wt%, 2 wt%, 2.6 wt% and 3 wt% in the PDMS matrix were studied. Table S1, Supporting Information, summarizes the dielectric constants of the composite dielectrics with various MWCNT weight ratios at different frequencies (10, 100, and 300 kHz). The characterization method is described in the Characterization and Measurement section. The dielectric constant increases with the increasing amount of MWCNT and reaches a maximum value of 13.61 at 2.6 wt%. When the MWCNT weight ratio increased further than 2.6 wt%, the dielectric constant slightly decreased. This can be attributed to the fact that the collapse of the microcapacitors formed in the PDMS matrix and the adjacent WMCNTs start to form ohmic conductive paths, which leads to the transition of the sensing material from the dielectric to conductivity.^[^ ^21^, ^22^ ^]^

Additionally, the impact of MWCNT weight ratios on the morphology of dielectric layers was explored. The surface and cross‐sectional morphologies of the 1 wt% and 2 wt% MWCNT/PDMS are shown in Figures S1 and S2, Supporting Information, respectively. The surface of the composite film with 1 wt% MWCNT was completely flat, while the film with 2 wt% MWCNT started to have small bumps with an average height of ≈10 µm. Scanning electron microscope (SEM) of the 2.6 wt% MWCNT/PDMS are shown in Figures 1d and 1e, respectively. It is obvious that the 2.6 wt% MWCNT/PDMS composite is rougher and possesses spontaneous wrinkles on the surface, which can also be observed in the optical images in Figure S2a,b, Supporting Information. According to Figure 1d and 1e, the thickness of the 2.6 wt% composite film is ≈160 µm, and the average height and width of the surface wrinkles are ≈45 and ≈280 µm, respectively. This also indicates that the size of the wrinkles increased with increasing MWCNT concentration. The irregular and randomly distributed wrinkles are attributed to the agglomeration of CNTs owing to the strong van der Waals forces, high aspect ratio, and low bending stiffness of the nanotubes.^[^ ^23^ ^]^ Furthermore, to illustrate the importance of these wrinkles on the sensor performance, we fabricated a flat film with a 2.6 wt% MWCNT/PDMS film via the blade coating technique, as shown in Figure S1a, Supporting Information.

**Figure 2:**
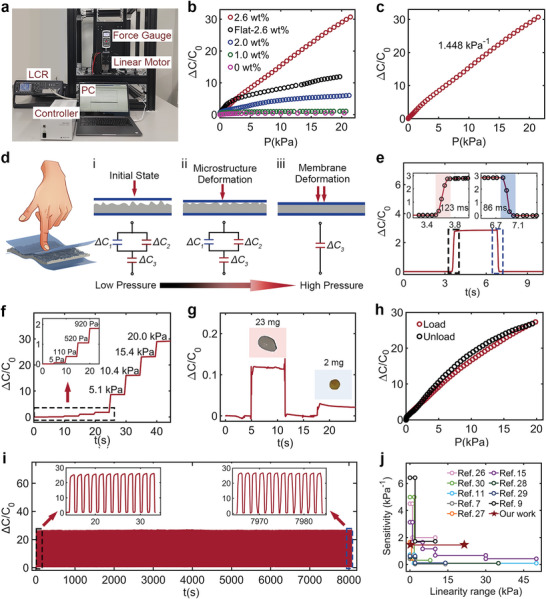
Sensing performances of the sensor. a) The experimental setup. b) Sensitivities of capacitive sensor with different MWCNT/PDMS ratios and surface morphology. c) Sensitivity of the sensor with 2.6 wt% MWCNT/PDMS dielectric layer. d) Sensing mechanism of the capacitive sensor with natural wrinkles. Equivalent circuit of the sensor i) without pressure, ii) when the sensor was subjected to low pressure, and iii) when the sensor was subjected to high pressure. e) Response and relaxation time of the capacitive sensor with 2.6 wt% MWCNT/PDMS when applied pressure was 0.9 kPa. f) Real‐time capacitive change rate of the sensor subjected to step pressure. g) Responses to ultralight objects (with the weight of 23 and 2 mg) and the limit of the pressure sensor, around 0.2 Pa. h) Hysteresis performance of the flexible pressure sensor. i) Stability of the sensor under the pressure of 17 kPa for 5000 cycles. j) Comparative study of the pressure‐sensing performance with respect to sensitivity and detection range between our sensor and recently reported ones.

The electromechanical performance of flexible capacitive sensors with various MWCNTs weight ratios was studied using the experimental setup shown in **Figure** **2**a. Figure 2b shows the relative capacitance change (*C* − *C*_0_/*C*_0_) in response to an applied pressure *P* for pressure sensors with different MWCNT ratios, where *C* and *C*_0_ are the capacitances in the loading and initiation states, respectively. The sensitivity of the capacitive pressure sensor is defined as *S = δ*(*C* − *C*_0_/*C*_0_)/*δP*. According to the sensitivity summary of the counterpart with 0, 1.0 wt%, 2.0 wt% and flat‐2.6 wt% MWCNTs (Table S2, Supporting Information), it is clear that the sensitivity increases with the increasing ratio of MWCNT owing to the increase in the dielectric constant and gradual formation of numerous wrinkles on the surface. The finite element analysis (Figure S3, Supporting Information) shows the intense stress concentration on the wrinkles, which plays a significant role in sensitivity improvement. However, the sensitivity of these sensors gradually decreased as the pressure increased, leading to poor linearity in the sensing range. This is unfavorable for practical applications because it requires an additional complicated readout circuit for data processing and conversion. Therefore, in this study, with the same MWCNT ratio of 2.6 wt%, the capacitive sensor based on a microstructured dielectric layer with randomly distributed wrinkles exhibited an ultrahigh sensitivity of 1.448 kPa^−1^ and excellent linearity (*R* ^2^ = 99.82%) over a broad pressure range of 0.005–21 kPa, as shown in Figure 2c. This sensitivity is 3.5 times higher than that of flat 2.6 wt% film and 145 times higher than that of pure PDMS in the low‐ to medium‐pressure range (2–21 kPa).

Based on the spontaneously wrinkled dielectric layer, excellent sensitivity, and linearity of the pressure sensor are achieved owing to the synergistic effect of spontaneous microstructures and the composite material. At the structural level, randomly distributed microstructures cause stress concentrations. At the material level, MWCNTs act as a high‐dielectric‐constant dopant, and the composite follows the percolation threshold theory. Generally, a capacitive pressure sensor is defined as the change in capacitance of a typical parallel plate capacitor with respect to the pressure change, as shown in Equation (1), where *ε*_0_, *ε*_r_, *A* and *d* are the permittivity of vacuum, relative dielectric constant of the dielectric material, overlapping area of the two electrodes and distance between the top and bottom electrodes, respectively. In our sensors, the overlapping area of electrode A is fixed and can be considered constant. Therefore, the capacitance is determined by *ε*_r_ and *d*. To clearly explain the change in *ε_r_* and *d* under pressure, the dielectric layer was divided into a wrinkled layer and a membrane layer, as shown in Figure 1d. To comprehensively illustrate the sensing mechanism of this linear pressure sensor, a simplified working flow chart and equivalent circuits are shown in Figure 2d.

(1) C=ε0εrε0εrAdAd

The wrinkled layer consists of two parallel‐connected capacitors, *C*_1_ and *C*_2_. *C*_1_ is a capacitor with a vacuum air dielectric layer and *C*_2_ is a capacitor with a wrinkled MWCNT/PDMS dielectric layer. The membrane layer was a capacitor with a flat MWCNT/PDMS film as the dielectric layer, represented by *C*_3_, which was connected to *C*_1_ and *C*_2_. In the initial state, no pressure is applied to the sensor, and three capacitors are formed and remain at the initial values, as shown in Figure 2d‐i. Once the sensor is subjected to external pressure, its capacitance increases instantly. In Figure 2d‐ii, when a small pressure is applied to the sensor, the irregular microstructures of the dielectric layer are compressed gradually, which induces a decrease in *d*. Because the microstructured layer consists of air and wrinkles, the Young's modulus is lower than that of the membrane layer. Therefore, a rapid increase *Δd* was observed in the microstructured layer, whereas  *Δd* of the membrane layer was negligible. According to the general Lichtenecker mixing rule,^[^ ^24^ ^]^ the relative dielectric constant *ε*_r_ of the dielectric layer is defined in Equation (2), where *ε*_Air_, *ε*_Composite,_ *V*_Air_ and *V*_Composite_ represent the permittivity of vacuum air, dielectric constant of the sensing material, volume percentage of air and MWCNT/PDMS composite, respectively. Here, *ε*_Air_ *=* 1 and *ε*_Composite_ for the 2.6 wt% MWCNT/PDMS composite is 13.61, as shown in Table S1, Supporting Information. When the sensor is compressed, air is gradually substituted for the MWCNT/PDMS, leading to an increase in *ε*_r_. Therefore, at stage ii, *ΔC*_1_ and *ΔC*_2_ induced by the deformation of the wrinkled dielectric layer determine the sensor response to pressure. *C*_3_ can be regarded as a constant and has a minor effect on the equivalent circuit. When the pressure increased further, as shown in Figure 2d‐iii, the irregular wrinkles collapsed and released the air. The microstructured layer becomes a flat membrane layer, and *ε*_r_ equals that of *ε*_Composite_. Therefore, the capacitance of capacitor *ΔC*_3_ determines the response of the entire sensor. The capacitance change completely depends on the deformation of the dielectric layer and the change in the dielectric constant,^[^ ^14^ ^]^ where a higher dielectric constant compensates for *Δd* owing to the high Young's modulus of the flat MWCNT/PDMS composite, resulting in a large capacitance change. Therefore, at stages ii and iii, the combination of wrinkled microstructures and a high dielectric constant enables the sensor with 2.6 wt% MWCNT/PDMS dielectric layer with high sensitivity and linearity. However, for other MWCNT concentrations, the mismatch between the microstructured morphology and dielectric constant compels them to exhibit a segmented response to pressure.

(2) $$\varepsilon_{r}=\varepsilon_{\mathrm{Air}}\;V_{\mathrm{Air}}+\varepsilon_{\mathrm{Composite}}V_{\mathrm{Composite}}$$

In addition to sensitivity and linearity, response time is another critical factor for various tactile applications. Figure 2e shows a fast response time of 123 ms and recovery time of 86 ms under a pressure of 0.9 kPa, which is slightly higher than the response time of human skin perception.^[^ ^25^ ^]^ Figure 2f shows the stepwise response of the sensor when a step pressure of 0–20 kPa was applied to the sensor. The inset shows the sensor response to a small pressure ranging from 0.005 to 1 kPa. Furthermore, it was found that the sensor response was not dependent on the temperature, as shown in Figure S4, Supporting Information. To comprehensively demonstrate the real‐time response of the sensor, it was attached to the index finger of a glove to distinguish the pressure when the human hand grasped and released a plastic cup, as shown in Figure S5, Supporting Information, where the capacitance increased when the amount of water increased. To further investigate the capability of perceiving subtle pressure beyond the normal detection range, our sensor was used to sense airflow using an air blower placed 10 mm above the sensor, simply indicating the ability to detect human respiration, as shown in Figure S6, Supporting Information. Concurrently, Figure 2g shows the sensor response to a domestic rice of 23 mg and an ultralight esculent millet of 2 mg (representing the pressure of ≈2.3 and ≈0.2 Pa, respectively), which indicates that compared with a 0.5 Pa detect limit of the commercial force gauge, the detection limit of our sensor can be as low as 0.2 Pa. These excellent performances demonstrate that our sensor possesses both high resolution and precision. In addition, the hysteresis test was carried out by loading and unloading pressure on the sensor, and a maximum hysteresis of 6.78% was achieved, as shown in Figure 2h. Moreover, the proposed sensor exhibited excellent robustness and durability, as shown in Figure 2i. No obvious degradation was observed under a pressure of 17 kPa for 5000 cycles, indicating the excellent long‐term stability of this pressure sensor. In Figure 2j, in terms of sensitivity and linearity, the comparison between our work and other recently reported sensors based on microstructured dielectric layers or high‐permittivity fillers was studied, and the details are listed in Table S3, Supporting Information.^[^ ^26^, ^27^, ^28^, ^29^, ^30^ ^]^ Most of the reported sensors consist of two or more linear ranges within the dynamic range. For instance, the porous Ecoflex‐MWCNT dielectric presented the highest sensitivity of 6.42 kPa^−1^ but a small sensing range of 2 kPa.^[^ ^9^ ^]^ Another copper calcium titanate‐wrapped hybrid sponge dielectric exhibited a wide sensing range of up to 125 kPa, but a low sensitivity of 0.026 kPa^−1^.^[^ ^11^ ^]^ Notably, our sensor exhibits a better trade‐off between sensitivity and linearity over a wide sensing range, which is realized using only a simple and low‐cost fabrication technique.

**Figure 3:**
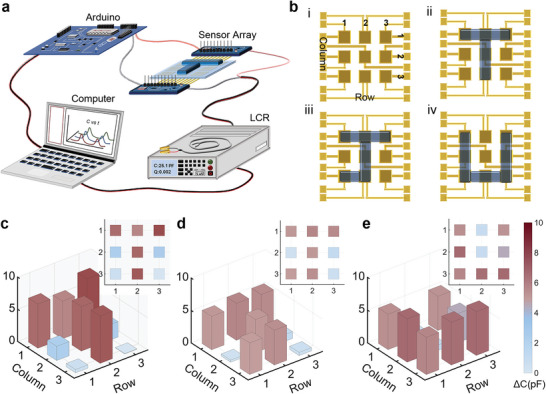
3 × 3 Sensor array for pressure distribution perception. a) Experimental setup. b) Schematic diagram of the sensor array with T, J, U‐shaped letter blocks placed on the top for pressure distribution perception. c–e) Histogram of the pressure distribution for three letters.

Additionally, based on the characterization of the performance, our pressure sensors can be further integrated into a sensor array with a PDMS layer encapsulated on the top for pressure distribution perception. Each pixel size was 5 mm × 5 mm, which can be further miniaturized in the future. **Figure** **3**a and Figure S7, Supporting Information, show the sequential data from nine pixels collected using a microprocessor (Arduino Uno) and multichannel collector (MUX). When the 3D printed “T”, “J” and “U”‐shaped letter blocks were positioned on the top of the sensor array (Figure 3b), the mappings of the capacitance change are presented in Figure 3c–e and each inset exhibits the vertical view of the corresponding histogram. It is obvious that the histograms agree with the profiles of the letter blocks, which further demonstrates the outstanding response to the pressure and provides a potential for various practical applications of tactile perception, such as physiological signal monitoring and the discrimination of material hardness.

### 2.2 Physiological Signals Monitoring

**Figure 4:**
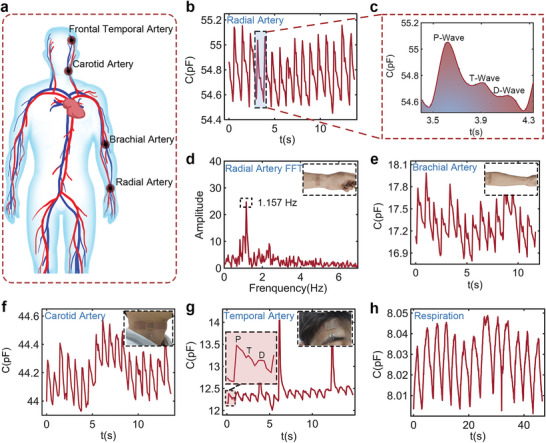
Flexible pressure sensor for continuous physiological signals monitoring. a) Schematic illustration of various possible monitoring artery positions. b) Real‐time radial arterial pulsation signal when the sensor is bonded on the wrist surface. c) A period of radial arterial signal extracted from the fourth waveform of the figure (b). d) Fast Fourier transformation for radial arterial pulsation signal to evaluate the frequency of pulsating. e) Real‐time brachial arterial pulsation when a young volunteer wears the sensor on the inside of the elbow. f) Real‐time carotid arterial pulsation signal and the shift are probably due to the respiration leading to the skin stretching. g) Real‐time temporal arterial pulsation signal, the higher peaks are attributed to the regular blinking. h) Real‐time respiration signal from the pressure sensor when the sensor is positioned ≈10 mm below the nostril. The inset of each subfigure shows the position of the sensor that is attached on the skin.

Cardiovascular disease is the leading cause of death and disability worldwide. The tactile perception of physiological signals is critical for cardiovascular disease prevention because it indicates the physiological functions of the circulatory and respiratory systems.^[^ ^31^ ^]^ As shown in **Figure** **4**, our pressure sensor can acquire various subtle artery pulsation signals from the radial artery of the wrist, brachial artery, carotid artery, and frontal temporal artery. The corresponding locations of these arteries (Figure 4a) can be found in several published studies.^[^ ^15^, ^32^ ^]^ To acquire the pulsation information, the flexible pressure sensor was attached to human skin with a preload with the aid of a commercial medical adhesive bandage.^[^ ^33^ ^]^ Figure 4b shows the real‐time output signal when the pressure sensor was placed over the radial artery of a 24‐year‐old healthy volunteer, and the fast Fourier transformation of this signal is shown in Figure 4d. According to the peak of 1.157 Hz, the pulse frequency was evaluated to be ≈70 beats/min, which falls within the normal range of a healthy person. Figure 4c presents the period of the waveform (the fourth radial arterial signal in Figure 4b) with a regression fitting that clearly distinguishes three typical peaks, including the percussion wave (P‐wave), tidal wave (T‐wave) and diastolic peak (D‐wave), which are consistent with the clinical monitoring.^[^ ^34^ ^]^ Similarly, Figures 4e and 4f show the results of the sensor tactile perception when our pressure sensor was placed on the volunteer's brachial artery and neck over the carotid arterial vessels. A waveform and pulse frequency similar to those of the radial artery signals was monitored. It should be noted that the low‐frequency fluctuation superimposed on the carotid arterial signal was due to the respiration of the volunteer. Simultaneously, the sensor was attached to the temporal artery, as shown in Figure 4g, and the real‐time output exhibited periodic temporal artery pulsation. Several high‐amplitude spikes are attributed to eye blinks, which induce a strain on the skin that is greater than the pressure of arterial pulsation. In addition to various pulsations, human respiration is also important for evaluating human health conditions.^[^ ^35^ ^]^ Similar to the detection of the breeze of air mentioned in the sensor characterization, the respiration of a human was also monitored. The sensor was placed 10 mm away from the human nose and the real‐time respiration signal was recorded, as shown in Figure 4h. With the above arterial pressure and respiration perception, our flexible sensor shows excellent potential for physiological signal monitoring, from detecting normal arterial pulsations to even subtle pulsations.

### 2.3 Hardness Tactile Perception Based on Soft Pneumatic Finger

**Figure 5:**
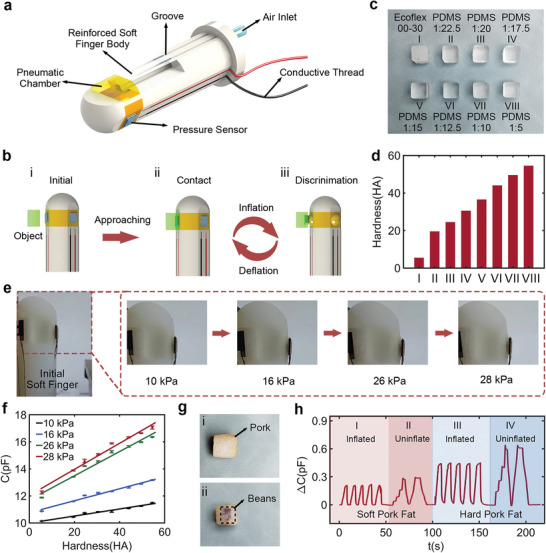
Soft robotic finger embedded with tactile sensor for hardness discrimination. a) Schematic of the soft finger with flexible pressure sensor attached to the inflation chamber. b) The schematic of a working cycle of the soft finger for the hardness discrimination. c) The photograph of 8 polymer blocks with different hardness. d) Measured hardness of polymer blocks by using a commercial hardness meter. e) Photographs of the chamber expansion under the different inflation pressures. f) Sensor response versus material hardness under different inflation pressures. g) Photographs of a block of pork fat without and with beans inside. h) Real‐time signals of the pressure sensors when the soft finger discriminates the raw fat with different hardness.

Hardness, as a physical property reflecting material mechanics, has a critical impact on safe and dexterous manipulation, especially in the era of intelligent devices with humanoid perceptions. Therefore, hardness discrimination has become significant tactile feedback in manipulation and has attracted considerable interest. In this study, we developed a soft pneumatic finger using multimaterial 3D printing technologies and integrated our pressure sensor to realize the tactile perception of hardness. **Figure** **5**a shows a schematic of the 3D printed finger and our pressure sensor (5 × 5 mm^2^) was attached to the soft pneumatic chamber. The detailed dimensions of the fingers can be found in Figure S8, Supporting Information. The finger had three inflation chambers, each connected to an airway. Two materials with different hardness were used to fabricate the finger: the inflation chamber was composed of soft material, and the remaining parts were made of reinforced hard material to restrict their expansion. Figure 5b presents the working cycle of a soft pneumatic finger with a regulated inflation pressure. First, the finger is in an initial state without inflation pressure. The object mounted on a linear motor gradually approached the finger, and the custom‐made experimental platform is shown in Figure S9, Supporting Information. Once the sensor attached to the finger contacts the object, inflation pressure is applied to the pneumatic chamber, thereby expanding and pushing the sensor toward the object. The pressure on the sensor results in a change in the capacitance. Here, a soft conductive thread (resistance < 1 Ω) was selected to transmit the output of the pressure sensor because it hardly affected the dynamic movement of the pneumatic chamber. Finally, when the inflation pressure was released, the finger and sensor recovered to their initial state and accomplished a hardness discrimination cycle.

For tactile hardness perception, we fabricated seven PDMS blocks with different hardness values by controlling the mixing ratio of the PDMS base and curing agent, including 1:5, 1:10, 1:12.5, 1:15, 1:17.5, 1:20 and 1:22.5. Additionally, we used Ecoflex 00–30 to fabricate a sample with lower hardness, as shown in Figure 5c. The hardness of the eight materials was measured using a commercial hardness meter, and the results are shown in Figure 5d. It is clear that with an increase in mixing ratios, the PDMS hardness linearly raises from 19.5 HA to 54.5 HA, as shown in Figure S10, Supporting Information. Compared to PDMS, Ecoflex 00–30 exhibits the smallest hardness of 5.5 HA. To explore the reliability of hardness discrimination based on pneumatic fingers, we investigated the sensor output five times by applying four different inflation pressures to drive the pneumatic chamber: i) 10, ii) 16, iii) 26, and iv) 28 kPa. Images of chamber expansion under different inflation pressures are shown in Figure 5e. The chamber gradually expanded when the finger was driven by an increase in inflation pressure. Before performing the hardness discrimination, as shown in Figure S11, Supporting Information, no interface was observed by running a working cycle without contacting objects and eliminating the influence of chamber expansion on the sensor response. Figure 5f presents the capacitance of the sensor as a function of hardness under four different inflation pressures, and a video was recorded (Movie S1, Supporting Information). For a specific inflation pressure, when the material hardness increased, the capacitance of the sensor increased linearly. This finding indicates that the combination of the pressure sensor and soft pneumatic finger can effectively distinguish material hardness. Meanwhile, the linear relationship (*R* ^2^ > 0.975) between the sensor capacitance and material hardness remained despite the change in inflation pressure. More importantly, the gradient of the fitting curves improved from 0.026 to 0.096 pF/HA with an increase in the inflation pressure from 10 to 28 kPa, which can be explained in Figure S12, Supporting Information. As shown in Table S4, Supporting Information, the hardness of the chamber increases when the inflation pressure increases. Thus, a harder chamber leads to an increase in the relative pressure between the sensor and objects, which induces a larger capacitance change and thereby achieves a higher sensitivity. However, considering the contradiction between the soft finger durability and hardness discrimination sensitivity, an inflation pressure of 26 kPa was selected as the optimal working condition, and repeatability was tested with 20 cyclic measurements, as shown in Figure S13, Supporting Information.

To further demonstrate the versatility of hardness perception based on the soft pneumatic finger and pressure sensor, we attempted to provide a preliminary evaluation of the difference between normal tissue and abnormal rectal tumors following digital rectal examination (DRE). Currently, DRE is one of the most direct diagnostic approaches to rectal cancer. It can be used to evaluate rectal canal health in terms of tumor hardness and rectal conditions (i.e., narrowed, swollen, eroded). Therefore, based on this simple and effective diagnosis, we employed pork fat with different hardness values to imitate the rectum in healthy and harder tumors. The hardness of the fat was tuned by embedding the beads, as shown in Figure 5g. Figure 5h shows the sensor response to soft fat (section I) and stiffened fat (section III) when the finger was driven by an inflation pressure of 26 kPa. Evidently, stiffened fat exhibits a larger capacitance change than soft fat, which preliminarily proves the feasibility of discriminating fats with different hardness values based on our finger and pressure sensor. Furthermore, the sensor response was studied when the object approached and then pressed the finger without inflation pressure in two ways: i) step‐by‐step with 0.5 mm increments; and ii) continuous motion at a speed of 1.5 mm s^−1^. The two methods employed the same compression displacement of 2 mm. The recorded capacitance changes in two different compressed ways are shown in sections II and IV of Figure 5h. It can be observed that the capacitance change for an object with a specific hardness is consistent despite the different pressing methods, which indicates that the discrimination is only determined by the compression displacement rather than the pressing process. These results provide a foundation for assessing rectal narrowing. For stepwise compression, the corresponding stepped capacitance change shows the ability to evaluate compression displacement. Thus, by combining the evaluation results under inflation pressures of 0 and 26 kPa, our pneumatic soft finger with a pressure sensor paves the way for clinical palpation and demonstrates the potential to screen for abnormal conditions of the rectum.

## 3 Conclusion

In this study, we propose a flexible capacitive pressure sensor with a spontaneous wrinkled MWCNT/PDMS dielectric layer fabricated via a simple spin‐coating process. The sensor exhibited an ultrahigh sensitivity of 1.448 kPa^−1^ and excellent linearity (*R* ^2^ = 0.9982), which was attributed to the synergistic effect of the wrinkled microstructures and high dielectric constant. Furthermore, owing to the stress concentration resulting from the spontaneously wrinkled microstructures, this sensor offers a number of high‐performance characteristics, including a low limit of detection, fast response and release, high durability, and robustness. These exceptional performances endowed this sensor with potential tactile perception for physiological health monitoring and pressure distribution. Additionally, to improve the safety and dexterity of various manipulations, such as robotic grasping and minimally invasive surgery, we developed a material hardness perception method based on a soft pneumatic finger and the proposed pressure sensor. Driven by a pneumatic finger, our sensor exhibited a linear response to material hardness, and the sensitivity was tunable via the inflation pressure of the finger. Furthermore, inspired by DRE for rectal cancer, the finger was used to successfully discriminate pork fat with different hardness. In the future, integrating a sensor with a compliant encapsulating structure into a pneumatic finger will be taken into consideration. This method can provide a foundation for replacing the human finger to realize more stable and accurate rectal health screening in the clinical setting.

## 4 Experimental Section

### Fabrication of Pneumatic Finger

The pneumatic finger was printed using a commercial multimaterial 3D printer (Objet500 Connex3, Stratasys). The capacitive pressure sensor was connected to a conductive silver thread using silver paste, and the sensor was attached to a plane wall connected to a soft pneumatic chamber using silicon paste. The geometric details of the soft fingers are shown in Figure S8, Supporting Information. The finger has three inflation chambers, and each connects to an airway to inflate these chambers. The finger body printed with hard rubber (Shore A 60) as a reinforced structure increased the pressure durability of the finger body and prevented the finger body from expanding under inflation. The soft pneumatic chamber was printed with soft rubber (Shore A 30), leading to faster expansion during inflation. Therefore, the cooperation of materials with different stiffnesses achieved partial expansion of the soft finger, which efficiently enhanced the pressure when the sensor contacted the blocks. It is noted that the silicon paste was used to seal the connection between the finger air passageways and flexible gas tubes. In addition, conductive silver fibers were bonded to the grooves of the finger body using silicon paste. Finally, a glossy finish was selected to ensure the smoothness of the prepared finger.

### Characterizations and Measurements

Optical and SEM images were captured using an Olympus optical microscope and a scanning electron microscope (ZEISS SIGMA). The force load was applied with a linear motor (PZG650‐R05AG‐E, Japan) that offers a gentle pressure gradient as a consequence of a minimum displacement change of 2 µm and a force gauge with a resolution of 0.0005 N (Mark‐10, USA.) The pressure was calculated using the relationship between the force and electrode area. The capacitance of the sensor output was measured using a high‐precision (around 10^−5^ pF) LCR meter (Keysight E4980AL) with a supply voltage of 1 V and a typical measurement frequency of 100 kHz. For the capacitance value acquisition of the sensor array, the MUX (CD74HC4067) by Arduino control was used for selecting the measurement channel, with MATLAB software assistant and the array data were acquired into a PC, facilitating the result process. For the hardness tactile perception experiment, the inflation pressure of the pneumatic chamber of the soft finger was controlled using a proportional valve (VEAB‐L26‐D7‐Q4‐V1‐1R1, Germany) with STM32. The feedback inflation pressure was monitored using a pressure meter (TM510; Tecman, China). A shore hardness meter (Type‐A) was used to measure the hardness of PDMS and Ecoflex.

### The Dielectric Constant Test of Dielectric Layer

The intrinsic dielectric constants of PDMS and MWCNT/PDMS composites with different doping concentrations were measured using an LCR meter. The MWCNT/PDMS samples were fabricated into flat membranes to prevent interference from surface microstructures. The effective dielectric constant of the hybrid composite was calculated using Equation (3), where *C* is the initial capacitance of a capacitor, *d* is the thickness of the composite, which is fixed at 1.15 mm. *A* is the area of the dielectric layer (9.44 × 9.48 = 89.5 mm^2^), which is identical to the electrode area.

(3) $$\varepsilon_{r}=\;Cd/A\varepsilon_{0}$$

## Conflict of Interest

The authors declare no conflict of interest.

## Supporting information

Supporting Information

Supplemental Video 1

## Data Availability

The data that support the findings of this study are available from the corresponding author upon reasonable request.

## References

[1] S. R. A. Ruth , V. R. Feig , H. Tran , Z. Bao , Adv. Funct. Mater. 2020, 30, 2003491.

[2] Y. Zang , F. Zhang , C.‐a. Di , D. Zhu , Mater. Horiz. 2015, 2, 140.

[3] K. Keum , J. Eom , J. H. Lee , J. S. Heo , S. K. Park , Y.‐H. Kim , Nano Energy 2021, 79, 105479.

[4] X. Cui , F. Huang , X. Zhang , P. Song , H. Zheng , V. Chevali , H. Wang , Z. Xu , iScience 2022, 25, 104148. 35402860 10.1016/j.isci.2022.104148 PMC8991382

[5] S. Li , K. Dong , R. Li , X. Huang , T. Chen , X. Xiao , Sens. Actuators, A 2020, 312, 112106.

[6] J. C. Yang , J.‐O. Kim , J. Oh , S. Y. Kwon , J. Y. Sim , D. W. Kim , H. B. Choi , S. Park , ACS Appl. Mater. Interfaces 2019, 11, 19472. 31056895 10.1021/acsami.9b03261

[7] Y. Luo , J. Shao , S. Chen , X. Chen , H. Tian , X. Li , L. Wang , D. Wang , B. Lu , ACS Appl. Mater. Interfaces 2019, 11, 17796. 31007008 10.1021/acsami.9b03718

[8] J. Hwang , Y. Kim , H. Yang , J. H. Oh , Composites, Part B 2021, 211, 108607.

[9] J. Choi , D. Kwon , K. Kim , J. Park , D. D. Orbe , J. Gu , J. Ahn , I. Cho , Y. Jeong , Y. Oh , I. Park , ACS Appl. Mater. Interfaces 2020, 12, 1698. 31825585 10.1021/acsami.9b20097

[10] D. M. Grannan , J. C. Garland , D. B. Tanner , Phys. Rev. Lett. 1981, 46, 375.

[11] A. Chhetry , S. Sharma , H. Yoon , S. Ko , J. Y. Park , Adv. Funct. Mater. 2020, 30, 1910020.

[12] L. Zhang , Z.‐Y. Cheng , J. Adv. Dielectr. 2011, 01, 389.

[13] B. Ji , Q. Zhou , M. Lei , S. Ding , Q. Song , Y. Gao , S. Li , Y. Xu , Y. Zhou , B. Zhou , Small 2021, 17, 2103312. 10.1002/smll.202103312 34585504

[14] J. Wu , Y. Yao , Y. Zhang , T. Shao , H. Wu , S. Liu , Z. Li , L. Wu , Nanoscale 2020, 12, 21198. 33057537 10.1039/d0nr06386j

[15] K.‐H. Ha , W. Zhang , H. Jang , S. Kang , L. Wang , P. Tan , H. Hwang , N. Lu , Adv. Mater. 2021, 33, 2103320. 10.1002/adma.202103320 34569100

[16] W. Yang , M. Xie , X. Zhang , X. Sun , C. Zhou , Y. Chang , H. Zhang , X. Duan , ACS Appl. Mater. Interfaces 2021, 13, 55756. 34780161 10.1021/acsami.1c17923

[17] Z. Zhang , J. Zhou , Z. Yan , K. Wang , J. Mao , Z. Jiang , Comput. Electron. Agric. 2021, 181, 105959.

[18] L. Zhang , F. Ju , Y. Cao , Y. Wang , B. Chen , Sens. Actuators, A 2017, 266, 197.

[19] S. Takamuku , G. Gómez , K. Hosoda , R. Pfeifer , 2007 IEEE 6th Int. Conf. Dev. Learn. 2007, 1.

[20] M. Xie , M. Zhu , Z. Yang , S. Okada , S. Kawamura , Nano Energy 2021, 79, 105438.

[21] P. Wei , X. Guo , X. Qiu , D. Yu , Nanotechnology 2019, 30, 455501. 31357189 10.1088/1361-6528/ab3695

[22] G. Liu , Y. Chen , M. Gong , X. Liu , Z.‐K. Cui , Q. Pei , J. Gu , C. Huang , Q. Zhuang , J. Mater. Chem. C 2018, 6, 10829.

[23] T. C. M , J. Pitchaimani , Appl. Math. Modell. 2022, 103, 68.

[24] H. Niu , S. Gao , W. Yue , Y. Li , W. Zhou , H. Liu , Small 2020, 16, 1904774. 10.1002/smll.201904774 31885133

[25] J. Yang , S. Luo , X. Zhou , J. Li , J. Fu , W. Yang , D. Wei , ACS Appl. Mater. Interfaces 2019, 11, 14997. 30869860 10.1021/acsami.9b02049

[26] Q. Su , Q. Zou , Y. Li , Y. Chen , S.‐Y. Teng , J. T. Kelleher , R. Nith , P. Cheng , N. Li , W. Liu , S. Dai , Y. Liu , A. Mazursky , J. Xu , L. Jin , P. Lopes , S. Wang , Sci. Adv. 7, eabi4563. 34818045 10.1126/sciadv.abi4563 PMC8612682

[27] C. Mu , J. Li , Y. Song , W. Huang , A. Ran , K. Deng , J. Huang , W. Xie , R. Sun , H. Zhang , ACS Appl. Nano Mater. 2018, 1, 274.

[28] Y. Wan , Z. Qiu , Y. Hong , Y. Wang , J. Zhang , Q. Liu , Z. Wu , C. F. Guo , Adv. Electron. Mater. 2018, 4, 1700586.

[29] S. Kang , J. Lee , S. Lee , S. Kim , J.‐K. Kim , H. Algadi , S. Al‐Sayari , D.‐E. Kim , D. Kim , T. Lee , Adv. Electron. Mater. 2016, 2, 1600356.

[30] Y. Kim , H. Yang , J. H. Oh , Mater. Des. 2021, 197, 109203.

[31] Z. Shen , F. Liu , S. Huang , H. Wang , C. Yang , T. Hang , J. Tao , W. Xia , X. Xie , Biosens. Bioelectron. 2022, 211, 114298. 35598556 10.1016/j.bios.2022.114298

[32] H. Ouyang , J. Tian , G. Sun , Y. Zou , Z. Liu , H. Li , L. Zhao , B. Shi , Yu. Fan , Yi. Fan , Z. L. Wang , Z. Li , Adv. Mater. 2017, 29, 1703456. 10.1002/adma.201703456 28863247

[33] P. Rwei , C. Qian , A. Abiri , Y. Zhou , E.‐F. Chou , W. C. Tang , M. Khine , Adv. Mater. Interfaces 2022, 9, 2200294.

[34] W. W. Nichols , Am. J. Hypertens. 2005, 18, 3S. 15683725 10.1016/j.amjhyper.2004.10.009

[35] X. Wang , Z. Liu , T. Zhang , Small 2017, 13, 1602790. 10.1002/smll.201602790 28306196

