# Supplementary material for: Ultrasensitive Linear Capacitive Pressure Sensor with Wrinkled Microstructures for Tactile Perception
Source: Adv Sci (Weinh). 2023 Mar 15;10(14):2206807. doi: 10.1002/advs.202206807 (PMC10190589; doi:10.1002/advs.202206807)
Supplement: Supplementary file 1 — Supporting Information [file ADVS-10-2206807-s002.pdf]

ADVANCED SCIENCE
Open Access

Supporting Information

for *Adv. Sci.*, DOI 10.1002/advs.202206807

Ultrasensitive Linear Capacitive Pressure Sensor with Wrinkled Microstructures for Tactile Perception

*Chunyu Lv, Chengcheng Tian, Jiashun Jiang, Yu Dang, Yang Liu, Xuexin Duan, Quanning Li, Xuejiao Chen and Mengying Xie**

Supporting Information

Ultrasensitive linear capacitive pressure sensor with wrinkled microstructures for tactile perception

Chunyu Lv, Chengcheng Tian, Jiashun Jiang, Yu Dang, Yang Liu, Xuexin Duan, Quanning Li, Xuejiao Chen, Mengying. Xie*

C. Lv, C. Tian, J. Jiang, Y.Liu, X. Duan, Q. Li, X. Chen, M. Xie

State Key Laboratory of Precision Measuring Technology and Instrument, College of Precision Instrument and Opto-electronics Engineering

Tianjin 300072, China

Email: mengying_xie@tju.edu.cn

Y. Dang

College of Artificial Intelligence, Nankai University

Tianjin 300350, China

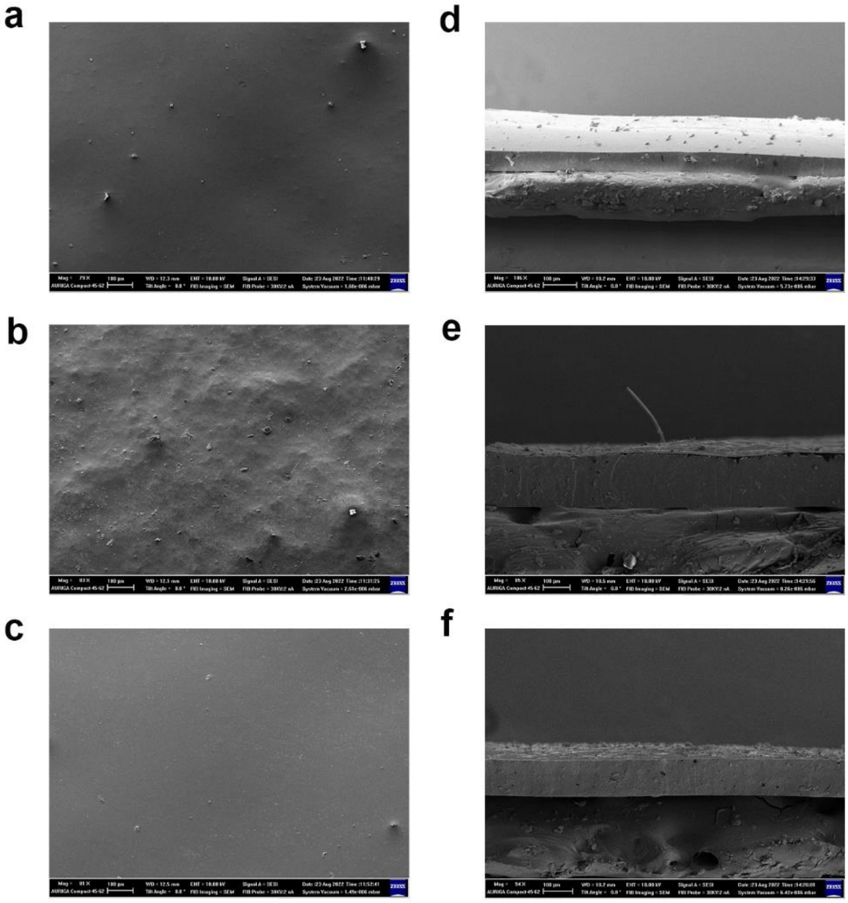

Figure S1. The SEM photograph of the surface of the MWCNT/PDMS dielectric layer with the MWCNT content of a) 2.6 wt% with blading treatment b) 2.0 wt% c) 1.0 wt%. The cross section of the dielectric layer with the MWCNT content of d) 2.6 wt% with scraping treatment e) 2.0 wt% f) 1.0 wt%.

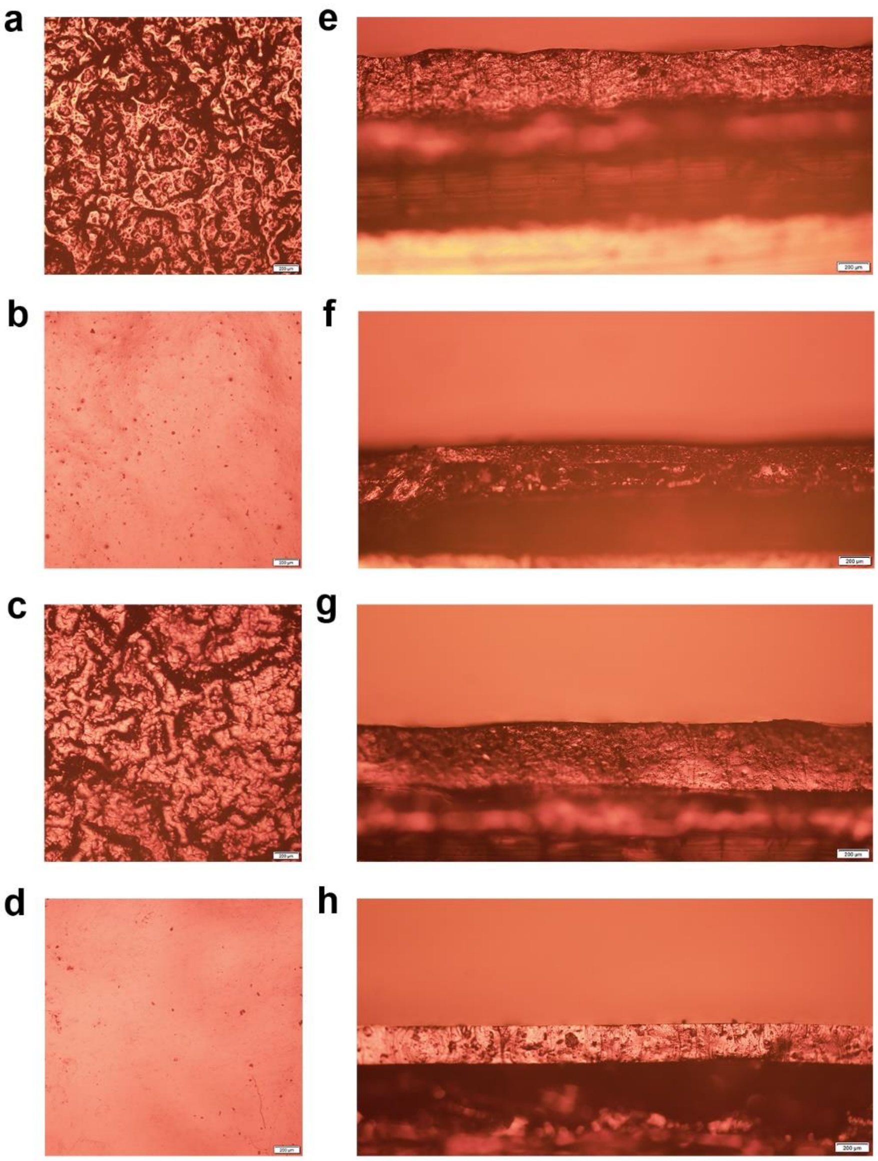

Figure S2. The optical photograph of the surface of the MWCNT/PDMS dielectric layer with the MWCNT content of a) 2.6 wt%b) 2.6 wt% with scraping treatment c) 2.0 wt% d) 1.0 wt%. The cross section of the dielectric layer with the MWCNT content of e) 2.6 wt% f) 2.6 wt% with scraping treatment g) 2.0 wt% h) 1.0 wt%.

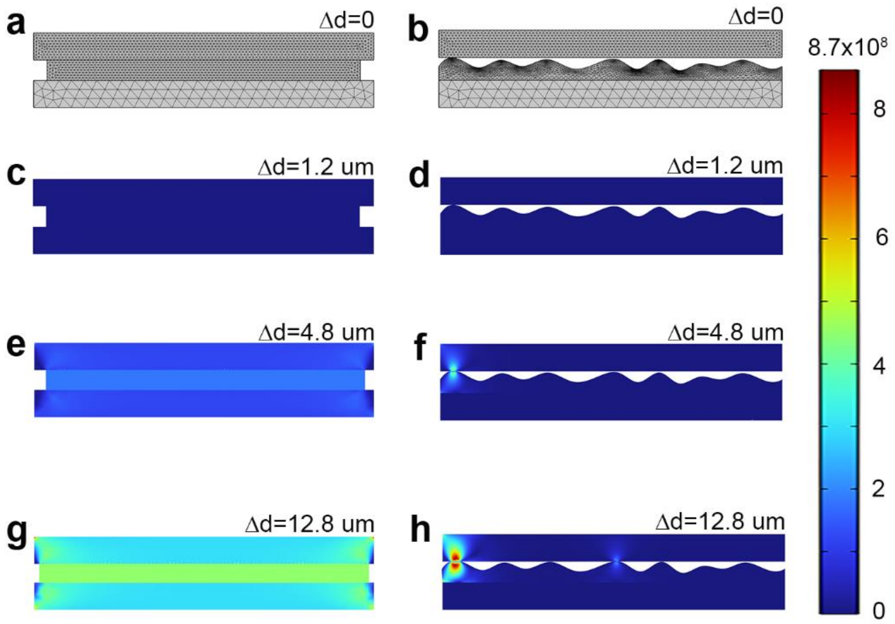

Figure S3. The FEA for the mechanics of two dielectric layers with different surface morphologies. a) b) The mesh for the flat dielectric layer and the microstructured dielectric layer with wrinkles morphology. The stress distribution and magnitude for the flat dielectric layer and the microstructured dielectric layer with wrinkles morphology c) d) under the displacement of 1.2 µm, e) f) 4.8 µm, g) h) 12.8 µm, respectively. The unit is N/m^2.

In this work, the COMSOL Multiphysics software was used for finite element analysis (FEA) to demonstrate the critical impact of a microstructured dielectric layer with randomly distributed wrinkles on the stress concentration, thereby evaluating the sensitivity of capacitive pressure sensors. Here, two dielectric layers with a thickness of ~160 µm were modeled, i.e, i) flat dielectric layer, ii) microstructured dielectric layer. Each dielectric layer was sandwiched by two plates. The upper plate was treated as the load for the dielectrics, while the bottom was the substrate of dielectrics. The schematic of the two models is shown in figure S3b and S3f, respectively. Note that the microstructured dielectric layer possessed wrinkles with a width of around 200 um and a varying height from 30 to 70 um, which represented the irregular wrinkled morphology of the MWCNT/PDMS composite due to the aggregation of MWCNTs. In addition, the precise meshing of the flat and microstructured

dielectrics is shown in figure S3a and S3b, respectively. As we can see, the tips of microstructures possess denser meshes, which is convenient for the model simulation with irregular morphology. For the mechanics analysis, a displacement increment was regarded as the constraint condition to compress the dielectric layers. It can facilitate model convergence in comparison with pressure as load, thus simplifying the simulation process.

In the beginning, the dielectric layers with different surface morphologies remain the initial state, as shown in figure S3c and S3d. Once the displacement was applied to the upper plates, the dielectric layer started to show deformation. As can be seen in figure S3e and S3f, two dielectric layers have been subjected to pressure due to the compression from the upper plate. Obviously, the dielectric layer with randomly distributed microstructures shows greater pressure, which is attributed to the decreased contact area between the upper plate and a dielectric layer resulting from the wrinkled morphology, which leads to stress concentration at the tips of wrinkles. With the further movement of the upper plate, although the two dielectric layers are suffered from increasing pressure, a similar relationship of stress distribution between the flat and microstructured dielectric layer still is observed, as shown in figure S3g and S3h. The flat dielectric layer still remains a uniform stress distribution. Expectedly, the microstructured dielectric layer possesses huger stress despite gradually contacting more wrinkled microstructures. Therefore, based on the above theoretical analysis, the wrinkled microstructures of dielectrics play an important role in the stress distribution increasing, thereby leading to sensitivity improvement of capacitive pressure sensors.

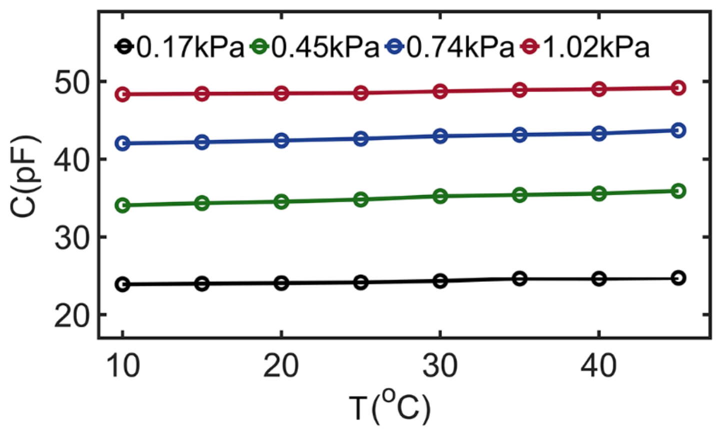

Figure S4. Temperature effect on the sensor performance under different pressure. Noted that the measurement temperature ranges from 10℃ to 45℃ in consideration of the various applications related to tactile perception.

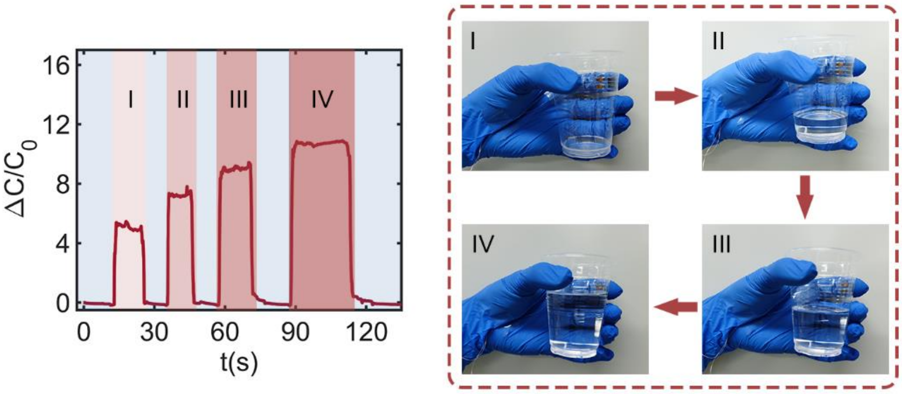

Figure S5. The glove attaching the sensor grasps the plastic cup filled with various weights of water.

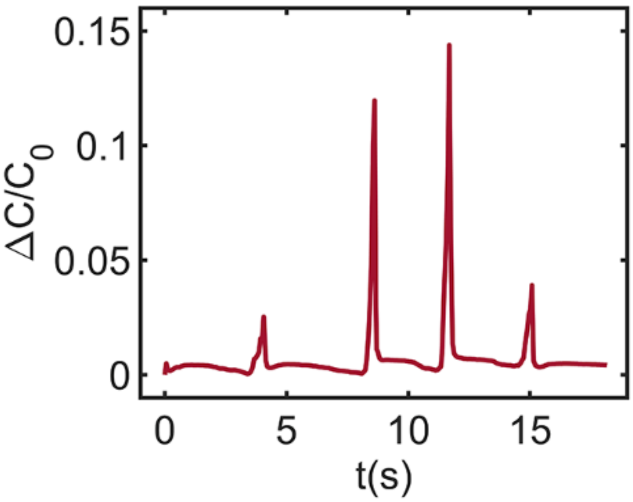

Figure S6. The sensor responses to the tiny blowing, and the different peaks represented the distance between the sensor and the air blower.

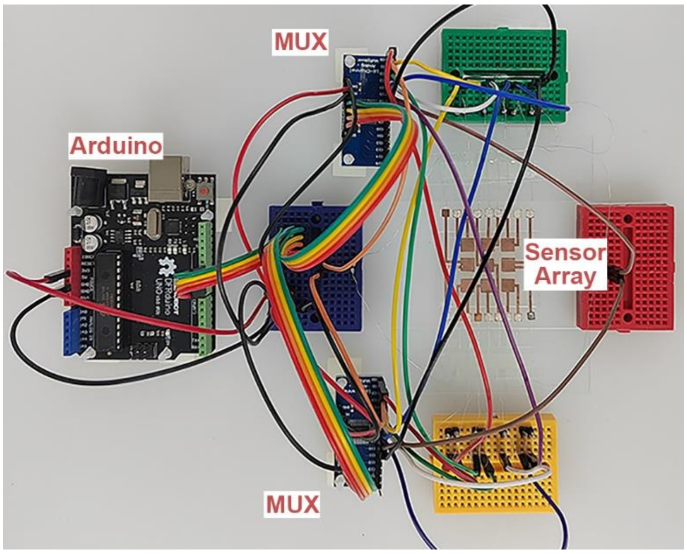

Figure S7. The setup for the capacitance acquirement of 3×3 sensors array.

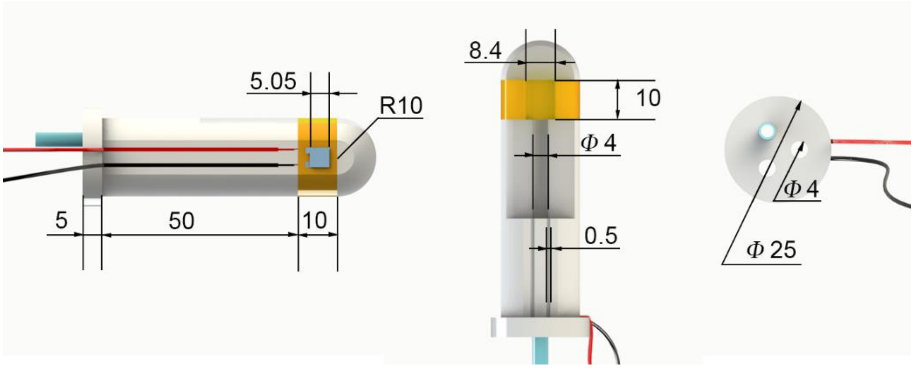

Figure S8. The detailed dimensions of the soft finger. The unit is mm.

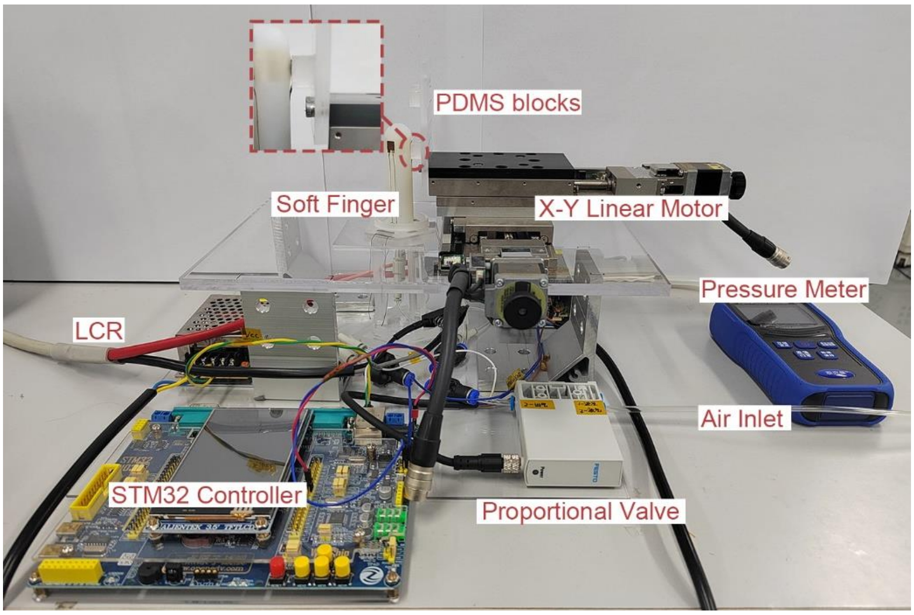

Figure S9. The setup for hardness identification based on the pneumatic finger, the inset shows the detail of contact between the finger and PDMS blocks.

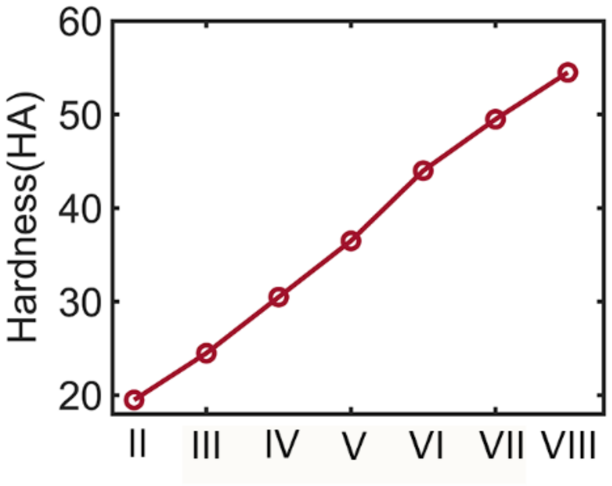

Figure S10. The linear relationship between the hardness and the ratio of PDMS base to curing agent. The labels II, III, IV, V, VI, VII, and VIII represent respectively the different ratio of curing agent to PDMS base, including 1:22.5, 1:20, 1:17.5, 1:15, 1:12.5, 1:10, 1:5.

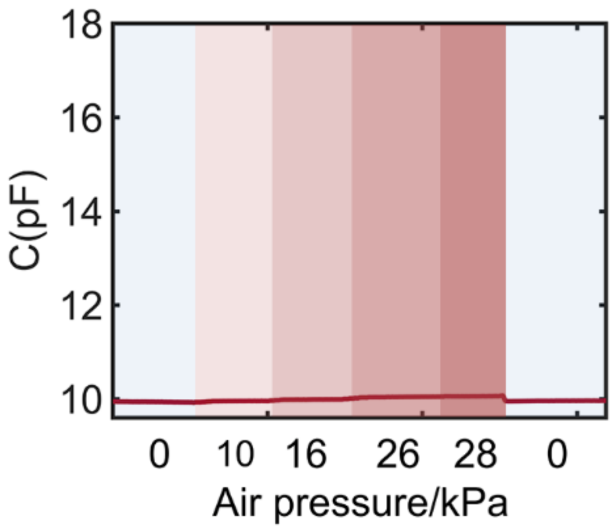

Figure S11. The capacitance change of the sensor attached to the pneumatic chamber of the soft finger along with the inflation pressure increasing and contactless with blocks. The darker red represents the higher inflation pressure.

Based on a soft pneumatic finger and the proposed pressure sensor, hardness discrimination can be realized by measuring the contact pressure between the sensor and the objects. The experimental results demonstrated the sensitivity to hardness is tunable via only controlling the inflation pressure of pneumatic chambers. However, to eliminate the hypothesis that the expansion of pneumatic chambers has an impact on the capacitance of the pressure sensor, a working cycle was carried out when the sensor attached to the finger was contacted without objects. Compared with the capacitance change during hardness discrimination, it shows a negligible capacitance variation, as shown in figure S11. This finding provides a proof for the independence of this pressure sensor when hardness discriminating.

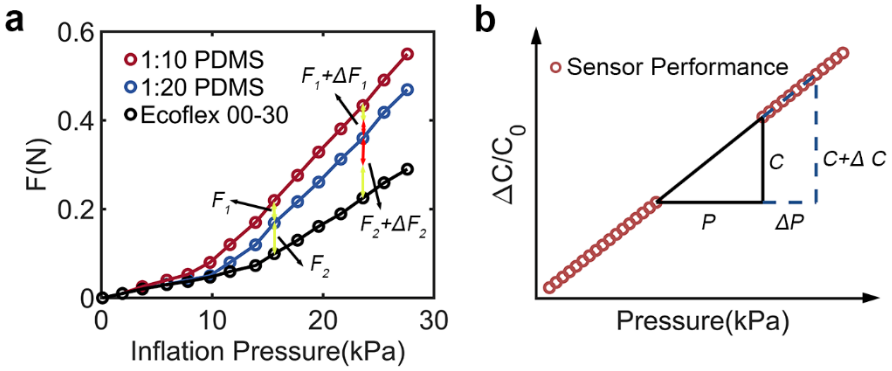

Figure S12. The schematic illustration of the increased sensitivity for hardness identification with the increasing inflation pressure of the soft finger. a) The relationship between the different inflation pressure and the reverse force from the hard objects. b) The schematic of reverse force and sensor capacitance increasing.

The hardness discrimination shows that the capacitance of the pressure sensor changed linearly to materials hardness in a low inflation pressure (10 kPa). Therefore, owing to the excellent linearity of our pressure sensor, the contact force (F) between the sensor and objects with eight different hardness linearly raises with the increasing hardness of objects. When the finger is driven by a higher inflation pressure (> 10 kPa), the pneumatic chamber becomes harder (can be proved in table S1), thus inducing an increment of force (ΔF) in comparison with that of low inflation pressure under the same hardness gradient, as shown in figure S12a. Consequently, in figure S12b, a more distinct capacitance change ($C+\Delta C$) is obtained. Therefore, for the same hardness gradient, with the inflation pressure increasing, the slope between the induced more apparent capacitance change ($C+\Delta C$) and the hardness gradient undoubtedly increases, thereby increasing the sensitivity to hardness.

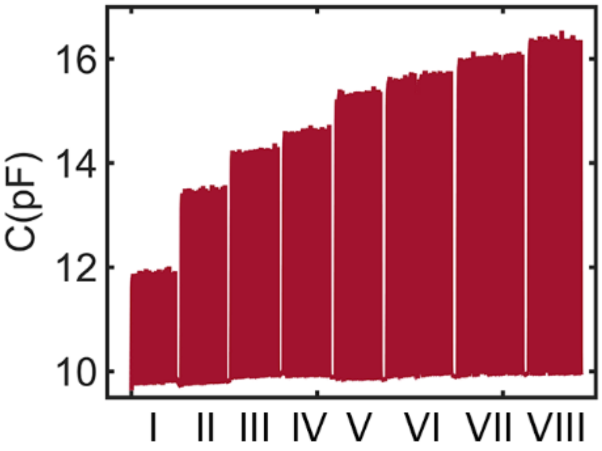

Figure S13. The sensor responses to the blocks with different hardness during 20 cyclic hardness identification tests. The label I, II, III, IV, V, VI, VII, and VIII represents respectively the hardness of 5.5 HA, 19.5 HA, 24.5 HA, 30.5 HA, 36.5 HA, 44 HA, 49.5 HA, 54.5 HA.

Table S1. The dielectric constant of MWCNT/PDMS composite with various MWCNT weight ratios at different frequencies and room temperature.

**Frequency (kHz)**	**CNT weight ratio (wt%)**				
	**0**	**1.0**	**2.0**	**2.6**	**3.0**
10	3.99	4.11	9.92	14.15	13.60
100	3.99	4.07	8.54	13.61	13.05
300	3.99	4.03	7.81	13.22	12.47

Table S2. The sensitivity of pressure sensors based on different MWCNT concentration dielectric layers.

MWCNT concentrate	Pressure range/kPa	Sensitivity/kPa^{-1}
0 wt%	0-2.5	0.14
	2.5-20.5	0.01
1.0 wt%	0-1.5	0.51
	1.5-20	0.02
2.0 wt%	0-8	0.44
	8-21.7	0.14
Flat-2.6 wt%	0-2.5	1.92
	2.5-20	0.41
2.6 wt%	0-21	1.448

In table S1, the sensitivity of sensors based on different MWCNT concentrations dielectrics is comprehensively summarized. When the dielectric material of the pressure sensor is only pure PDMS, it possesses 0.14 kPa^{-1} and 0.01 kPa^{-1} in the pressure regimes of 0-2.5 kPa and 2.5-20.5 kPa, respectively. As the conductive filler of MWCNTs doping, it is clear that the sensitivity increases and shows a raising trend with the increasing ratio of MWCNT. This is attributed to the gradual formation of numerous wrinkles, which resulted from the MWCNT aggregations due to the strong Van der Waals forces. Moreover, the dielectric constant also increases with the ratio of MWCNTs raising based on the percolation threshold, which also contributes to sensitivity improvement. Specifically, the sensitivity of the sensor with 1 wt% MWCNT is approximately 0.51 kPa^{-1} (< 1.5 kPa) and 0.02 kPa^{-1} in the pressure range of 1.5-20 kPa. The sensitivity of sensor with 2 wt% MWCNT is 0.44 kPa^{-1} in 0-8 kPa and 0.14 kPa^{-1} in 8-21.7 kPa, respectively. Similarly, the sensor with bladed flat 2.6 wt% MWCNT/PDMS layer exhibits two linear regions, i.e., the sensitivity is 1.92 kPa^{-1} when the pressure is below 2.5 kPa, and is 0.41 kPa^{-1} in the pressure range of 2.5-20 kPa. As mentioned above, most of the reported sensors possess multiple linear regimes and each with a corresponding sensitivity

value. This is unfavorable for practical applications since it requires an additional complicated readout circuit for data processing and conversion. Therefore, a linear sensor is indeed attractive for practical applications. In this work, with the same MWCNT ratio of 2.6 wt%, the capacitive sensor based on a microstructured dielectric layer with randomly distributed wrinkles exhibits an ultrahigh sensitivity of 1.448 kPa^{-1} and excellent linearity (R^2 = 99.82%) in a broad pressure range of 0.005-21 kPa. This sensitivity is 3.5 times higher than flat 2.6 wt% film and 145 times higher than pure PDMS in the low- to the medium-pressure regime (2-21 kPa).

Table S3. The performance of pressure sensors of recently reported studies based on various fillers and microstructured dielectric layers.

Materials	Structures	Sensitivity/kPa^{-1}	Range/kPa	LOD/Pa	Ref.
PDMS	Tilted pillars	0.42	0-2	~	7
		0.04	2-14		
Ecoflex/MWCNT	Porous nanocomposite	6.42	0-2	~	9
		1.72	2-10		
PU/CCTO	Porous membrane	0.73	0-1.6	~	11
		0.135	1.6-22.8		
		0.06	22.8-50		
Ecoflex/CNT/PMMA	Porous nanocomposite	3.13	0-1	0.07	15
		1.65	1-5		
		1.16	5-10		
		0.68	10-30		
		0.43	30-50		
PVDF-HFP	Pyramids	4.5	0-1	0.2	26
		2	1-10		
PDMS/CCTO	Porous sponges	1.66	0-0.64	~	27
		0.46	0.64-1.4		
PDMS/AgNWs	Inspired cones	1.194	0-2	0.8	28
		0.3	2-7		
		0.077	7-15		
PDMS	Porous membrane	0.63	0-2	~	29
		0.125	2-14		
PDMS/FC-42,3M	Porous cones	4.99	0-1.7	~	30
		0.32	1.7-8		
PDMS/MWCNT	Spontaneous wrinkles	1.448	0-21	0.2	Our work

Table S2 summarizes key characteristics of the reported studies, including sensing materials, the microstructured dielectric layer, sensitivity in different pressure regimes, and the limit of detection. As can be seen, various microstructures of dielectrics have endowed pressure sensors with excellent performance, especially high sensitivity. However, when the pressure

increases further, the obvious attenuation of sensitivity is a common problem, thus leading to the difficulty of signal processing. Moreover, most of the sensors show a relatively narrow sensing range, which also limits their practical application. For example, ref.13 fabricated a porous Ecoflex-MWCNT composite via a sugar template, the synergistic effect of porous elastomer and the percolation of conductive fillers concentrated on the high sensitivity of 6.42 kPa^{-1} and 1.72 kPa^{-1} in a range of 0-2 and 2-10 kPa, respectively. The Ref. 9 also proposed a pressure sensor with a sensitivity of 4.99 kPa^{-1} in the range of 0-1.7 kPa, which was realized by a microstructured dielectric layer combined microcones with hieratical micropores. Similarly, the segmented response to pressure caused a decrease in sensitivity with the pressure being beyond 1.7 kPa, which demonstrated poor linearity of the sensor. Therefore, it still is a critical challenge to fabricate a pressure sensor with high sensitivity and linearity, simultaneously. In our work, a spontaneous wrinkled dielectric layer via a simple spin-coating process was proposed. The synergistic effect of wrinkled microstructures and the percolation of MWCNTs fillers enabled our sensor to high sensitivity of 1.448 kPa^{-1} in a broad pressure regime of 0.005-21 kPa. More importantly, excellent linearity (R^2=0.9982) also was realized, which provides a new method for the trade-off between the sensitivity and linearity of pressure sensors.

Table S4. The hardness of soft finger under different inflation pressure.

**Inflation Pressure/kPa**	0.1 kPa	9.9 kPa	15.8 kPa	19.6 kPa	25.9 kPa
**Hardness/HC**	1	3.5	6.5	8	9
